# A Superficial Anatomical Variation in the Radial Artery Renders It Unsuitable as a Graft in Coronary Revascularization

**DOI:** 10.7759/cureus.56298

**Published:** 2024-03-16

**Authors:** Christos Voucharas, Angeliki Vouchara, Dimitrios Bismpos

**Affiliations:** 1 Department of Cardiothoracic Surgery, Aristotle University of Thessaloniki, Thessaloniki, GRC; 2 Department of Surgery, Aristotle University of Thessaloniki, University General Hospital of Thessaloniki (AHEPA), Thessaloniki, GRC; 3 Department of Cardiology, Interbalkan Medical Center, Thessaloniki, GRC

**Keywords:** coronary artery bypass grafts, radial artery for cabg, superficial radial artery, radial artery anatomic variations, radial artery anatomy

## Abstract

In our experience, employing a radial artery in combination with an internal thoracic artery under specific conditions represents a superior option compared to using a vein graft in coronary artery surgery. Additionally, this combination is a remarkable alternative to utilizing bilateral thoracic arteries.

We had planned the left radial artery as the second target graft in two patients undergoing coronary artery bypass surgery; the left internal thoracic artery to the left anterior descending branch would be the main graft in both patients.

Anatomic variation of the radial artery, which presented as a superficial radial artery in both patients, led us to forego the use of the radial artery graft. This decision aimed to ensure sufficient blood supply to the palmar arch and prevent any potential inadequacy in the length of the coronary graft.

The occurrence of this variation is exceptionally rare, accounting for approximately 0.02% based on our experience with radial artery harvesting. Furthermore, globally, the documentation of photographs depicting a superficial radial artery is even more infrequent.

## Introduction

Half a century has passed since the initial utilization of the radial artery (RA) as a graft in coronary artery bypass surgery and one-third of the century since revival and then selective use of RA as a credible second or third target conduit in such procedures [1,2].

In contemporary times, it has been demonstrated that utilizing the radial artery as a secondary graft following the left internal thoracic artery (LITA) for coronary artery bypass grafting (CABG) to the left anterior descending (LAD) artery is both effective and safe. Under specific conditions, the outcomes and patency rates surpass those observed in saphenous vein grafting [3].

Currently, it is widely accepted that it is justifiable to employ as many arterial grafts as possible for each eligible candidate unless a specific contraindication is present. Surgeons argue that using the LITA along with the RA is superior to utilizing bilateral internal thoracic arteries. The guidelines from the European Society of Cardiology (ESC) and the European Society for Cardio-Thoracic Surgery (ESCTS) in 2017 and 2018 have assigned a class 1A designation to the RA, definitively establishing it as the second preferred conduit for CABG [4,5].

An infrequently observed factor that might hinder the use of the RA is an unusual anatomical variation in its path and branching structure, potentially impacting blood flow to the palm arch or the graft's length. Typically, variations in the anatomy of the RA are uncommon and typically do not influence its appropriateness for CABG [3].

## Case presentation

Following the previously outlined criteria, we devised a plan for two male patients, aged 65 and 62 years, both experiencing stable angina and diagnosed with critical coronary artery disease. The first patient, with a 90% stenosis in the obtuse marginal (OM) branch, was scheduled to undergo a procedure involving a LITA on LAD, a left RA on the OM, and a vein graft on the first diagonal (D1) branch. The second patient, presenting a 95% stenosis in the right coronary artery (RCA) just proximal to the crux cordis, was planned for a LITA on LAD, a left RA on the posterior descending artery (PDA), and a vein graft on D1.

In the case of the first patient, the presence of diabetes played a significant role in the decision to exclude the use of a second internal thoracic artery. For the second patient, the feasibility of a right internal thoracic artery (RITA) graft was limited due to its insufficient length for anastomosis on the PDA.

The right RA was the site of catheterization for coronary angiography in both patients. Following a positive modified Allen test, the left RA was prepared. Because of the anatomical variation discovered during the RA harvesting process, its utilization was abandoned. Instead, an additional saphenous venous (SV) graft was employed as a replacement.

**Figure 1:**
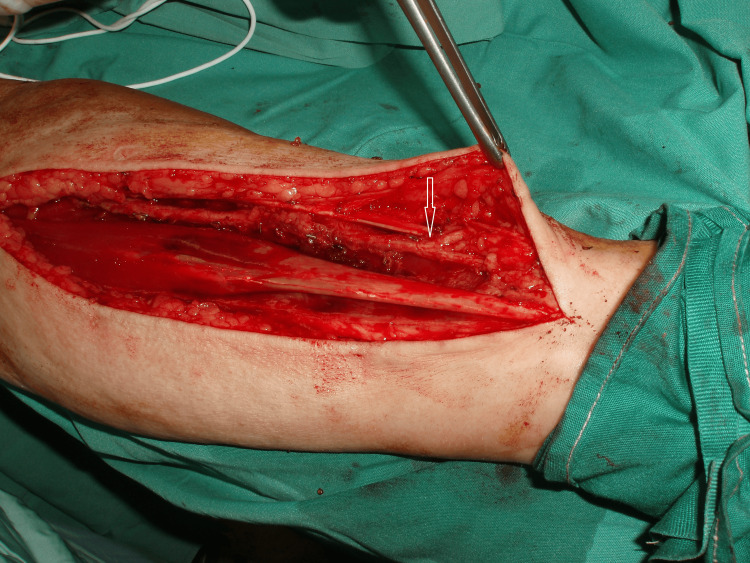
The radial artery follows a superficial route at the distal quarter of its course, highlighted by the arrow

In Figure 1, the left RA of the first patient is visible. Towards the distal quarter of the forearm, the radial artery emitted a dorsal superficial branch that traveled over the brachioradialis tendon and the extensor tendons of the thumb. The deep radial artery followed the typical path of the true radial artery. The decision was made to refrain from using the radial artery, indicating that in certain cases, this variant artery may serve as the dominant source of blood flow to the hand.

**Figure 2:**
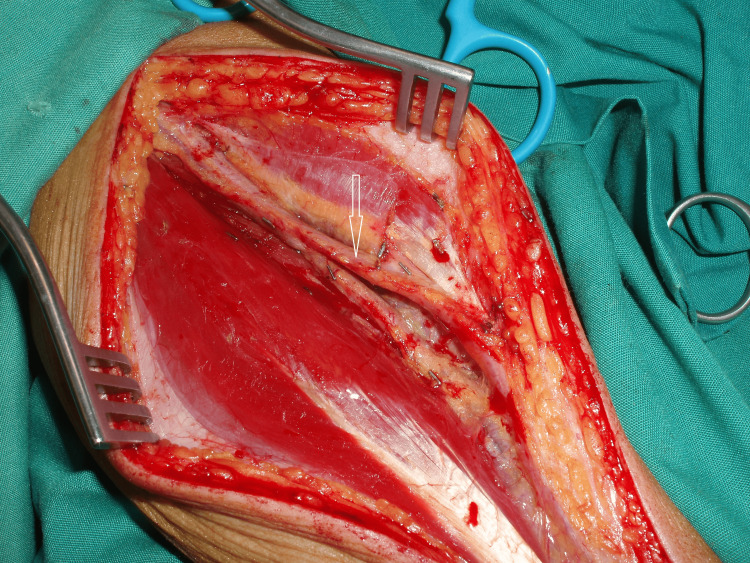
At the middle of its way, the radial artery passes superficially, highlighted by the arrow

In Figure 2, the photograph captures the left RA of the second patient. Along the midsection of the forearm, the left RA traversed dorsally around the superficial aspect of the brachioradialis muscle, while its deep branch followed the customary path of the RA to the wrist. However, the RA was deemed unsuitable as an aorta-to-PDA conduit due to its inadequate length.

## Discussion

Ordinarily, the radial artery courses along the radial aspect of the anterior compartment of the forearm, passing beneath the brachioradialis muscle and tendon, and lies lateral to the flexor carpi radialis tendon. Distally, the radial artery lies on the surface of the radius; it then progresses dorsally deep within the 'anatomical snuff box' between the two heads of the first dorsal interosseous muscle and emerges anteriorly. At the end of its course, the radial artery anastomoses with a deep branch of the ulnar artery at the base of the fifth metacarpal, contributing to the formation of the deep palmar arch [6-8].

To serve as a coronary bypass graft, the RA is incised proximally just peripheral to the recurrent RA and distally close to the wrist. It is important to retain as many branches around the wrist as feasible. With a length of around 20 centimeters, the radial artery is well-suited for use in coronary procedures, including those involving the most distal coronary targets [5].

Utilizing the radial artery for an aorta-to-coronary bypass graft is not recommended in specific situations. These include instances where the coronary stenosis is less than 70%, or the radial artery exhibits calcification, spasm, or traumatic injury (resulting from prior arterial punctures for procedures such as blood gas sampling or catheterization, as well as transradial angiography). Additionally, factors such as small size, late bifurcation of the brachial artery, and other anatomical variations in the course and branching pattern of the radial artery should be considered [3,9].

The superficial radial artery represents an anatomical variant in which the radial artery or one of its branches courses superficial to the lateral tendons of the anatomic snuffbox (the tendons of the hand's abductor pollicis, extensor pollicis longus, and extensor pollicis brevis muscles) [10,11].

The particular variation is exceptionally uncommon; this occurs 1% of the time [12]. Fewer than 25 cases have been described in the literature since its initial report in "Quain's Elements of Anatomy", a book published by Wood et al. in 1878 [11-13].

Moreover, visual documentation of this variant is even scarcer, with only a handful of photographs having been published [8,14].

We encountered and captured images of two instances of a superficial RA in a collection of 1003 radial arteries harvested by us in the last 20 years (0.2%). These occurrences prompted adjustments to our procedural approach.

When scheduling a coronary bypass surgery, it's not always feasible to perform routine angiography of the forearm in every patient. Additionally, there is a risk of harm to the distal end of the radial artery during angiography. The surgeon can make a continuous incision in the skin and subcutaneous tissue of the forearm from one end to the other, allowing for a complete view of the radial artery's course. However, in cases where the radial artery is atheromatous (commonly observed near the proximal edge), a staged incision will quickly reveal its unsuitability. Furthermore, less invasive radial artery harvesting techniques, either endoscopic or tunneling [15], can be detrimental to a variant radial artery.

An anomalous superficial course of the radial artery at the anatomical snuff box may be significant from a clinical standpoint, particularly for vascular and plastic surgeons. They often utilize the radial forearm flap, which is based on the radial artery, to reconstruct various anatomical structures in the head and neck [16].

Additionally, the superficial arteries of the upper extremity may often be mistaken for veins; injecting specific medication into an artery may be harmful [9].

## Conclusions

We present two cases of anomalous course of a radial artery indented to be a graft for coronary artery bypass surgery; the variant modified the intervention that had been planned. Variations in radial artery anatomy are frequently encountered. However, the presence of a superficial radial artery is particularly remarkable. When utilizing the radial artery for coronary bypass grafting, procedural challenges may arise, with potential differences in failure patterns depending on the specific anomaly present.
